# The Multifunction of TRIM26: From Immune Regulation to Oncology

**DOI:** 10.2174/0109298665311516240621114519

**Published:** 2024-07-02

**Authors:** Jialai Zou, Kaiyi Niu, Tao Lu, Jianxun Kan, Hao Cheng, Lijian Xu

**Affiliations:** 1 Department of General Surgery, The Second Affiliated Hospital of Nanjing Medical University, Nanjing 210011, China

**Keywords:** TRIM26, ubiquitination, viral infections, inflammation, carcinogenesis, physiological function, pathological function

## Abstract

Ubiquitination, a crucial post-translational modification, plays a role in nearly all physiological processes. Its functional execution depends on a series of catalytic reactions involving numerous proteases. TRIM26, a protein belonging to the TRIM family, exhibits E3 ubiquitin ligase activity because of its RING structural domain, and is present in diverse cell lineages. Over the last few decades, TRIM26 has been documented to engage in numerous physiological and pathological processes as a controller, demonstrating a diverse array of biological roles. Despite the growing research interest in TRIM26, there has been limited attention given to examining the protein's structure and function in existing reviews. This review begins with a concise overview of the composition and positioning of TRIM26 and then proceeds to examine its roles in immune response, viral invasion, and inflammatory processes. Simultaneously, we demonstrate the contribution of TRIM26 to the progression of various diseases, encompassing numerous malignancies and neurologic conditions. Finally, we have investigated the potential areas for future research on TRIM26.

## 1 INTRODUCTION

In the presence of a series of specialized enzymes, ubiquitination modifies target proteins within the cell using ubiquitin, a protein molecule consisting of 76 amino acid residues [1]. Ubiquitination modification consists of a three-enzyme cascade catalyzed by E1 (ubiquitin-activating enzyme), E2 (ubiquitin-conjugating enzyme), and E3 (ubiquitin ligase), which results in the formation of a stable isopeptide bond between the C-terminal carboxyl group of the ubiquitin molecule and the ε-amino group of Lys of the target protein [2]. In this process, E1 activates ubiquitin by binding to the Cys residue in the tail of the ubiquitin molecule in the same manner as adenosine triphosphate and transfers it to E2, where it finally co-recognizes specific target proteins with a specific E3. This results in the formation of an isopeptide bond between the glycine at the carboxy-terminal of the ubiquitin and a Lys of the substrate. Since E3s tightly control the efficiency and substrate specificity of the ubiquitination reaction, they are key components of this cascade reaction [3]. In addition, UFD2 was initially found to be able to reinitiate polyubiquitination after the completion of the three-enzyme cascade, and the researchers referred to this unique type of ubiquitin ligase as E4 [4]. Subsequent studies have revealed that the E4 lyases may also be involved in the formation of the E3-E4 or E4-substrate complexes to coordinate the transfer of ubiquitin from the E2 substrates [5]. Multiple research studies have demonstrated the significance of ubiquitination in various physiological and pathological processes [6]. Ubiquitination can achieve a wide range of cellular functions through protein hydrolysis and non-protein hydrolysis effects [7]. In gastric cancer, the activation of the AKT signaling pathway, regulated by miR-195-5p and facilitated by TRIM14, enhances migration and invasion by promoting Epithelial-mesenchymal Transition (EMT) [8]. Similarly, the ubiquitin ligase, Parkin, is able to target damaged mitochondria, activated by phosphorylation of the mitochondrial kinase PINK1 (PTEN-induced putative kinase 1), which ubiquitinates a variety of mitochondrial outer membrane proteins and initiates autophagy in damaged organelles [9, 10]. During the insulin resistance phenomenon induced by aging, cancer, and diabetes, the body leads to muscle catabolism by inducing the expression of the muscle-specific ubiquitin ligases Atrogin-1 and MuRF1 [11].

The Tripartite Motif-containing (TRIM) protein family consists of a RING structural domain at the N-terminal, one or two B-box structural domains, a Coiled Coil (CC) structural domain, and a highly variable carboxyl-terminal structural domain. The RING-finger structural domain is a zinc-binding motif with E3 activity, leading to the identification of most TRIM family proteins as E3 ubiquitin ligases. So far, only eight proteins in the TRIM family have been identified as lacking the RING structural domain [12], but part of them still have activity like classically structured E3 enzyme, such as TRIM16 [13]. Target protein recognition involves the B-box structural domains, which consist of two types: B-box1 and B-box2. These domains typically comprise small peptide sequences with finger-like protrusions. The CC structural domains are involved in TRIM homodimerization or oligomerization [14]. The C-terminal structural domains, which include the PRY structural domain, the SPRY structural domain, and others, determine the functional specificity of TRIM proteins and categorize them into 11 subfamilies [15, 16]. A large number of studies have shown that TRIM family proteins play important roles in a variety of physiological and pathological processes. Within the realm of innate immunity, TRIM5α has the ability to combat an array of viruses, encompassing retroviruses and lentiviruses. It has the ability to destroy viruses directly through the SPRY structural domain, and it also can hinder viral infection by triggering the NF-κB signaling pathway through TAK1 (Transforming growth factor-β Activated Kinase-1) activation [17, 18]. On the other hand, certain TRIM proteins are susceptible to viral immune evasion. For instance, the influenza A virus Nonstructural Protein 1 (NS1) has the ability to engage with TRIM25, hindering the subsequent ubiquitination of the viral RNA receptor RIG-I and the production of type I Interferon (IFN) [19]. TRIM proteins associated with the immune system may also have a significant impact on inhibiting the development of cancer and its advancement *via* the NF-κB or IFN signaling pathways [12]. A considerable number of neurodegenerative diseases are triggered by the production of aberrant aggregates of proteins and TRIM family proteins function by promoting the degradation of wrongly aggregated proteins by mediating ubiquitination, autophagy, and other pathways [20]. TRIM family proteins also play a role in metabolism-related fatty liver disease and diabetes mellitus [21, 22].

Although great progress has been made in the study of the TRIM family, its mechanism of action still deserves further exploration. There is little or no report on TRIM26 to the best of our knowledge. First, we have described the structure, expression profile, and localization of TRIM26 in cells, followed by a description of the regulatory role of TRIM26 in immunity, inflammation, and viral infection. At the same time, we have illustrated the role of TRIM26 in disease development, including tumors and neurological disorders. Finally, we have discussed our outlook and future research directions of TRIM26 with the aim of providing new ideas for the development of novel antiviral drugs and enriching tumor therapeutic modalities.

## 2 STRUCTURE, EXPRESSION, AND SUBCELLULAR LOCATION OF TRIM26

**Figure 1:**
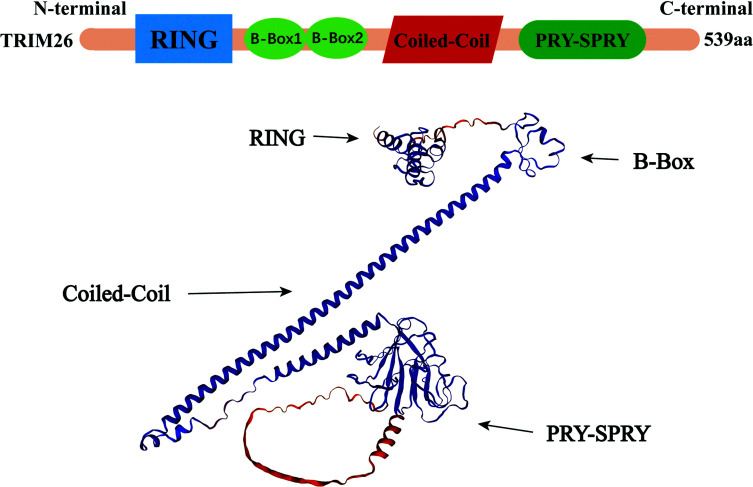
Structural model diagram and three-dimensional schematic structure of TRIM26.

TRIM26, also known as RNF95, ZNF173, is a 62 kDa protein containing 539 amino acid residues, encoded by the Major Histocompatibility Complex (MHC) class I region on chromosome 6, localized on 6p21.33-6p22.2, consisting of 9 introns and 10 exons spanning a length of 3518 bps [23–25]. TRIM26 has a similar RBCC structure, namely a RING finger encoding E3 ubiquitin ligase activity, a B-box, and a coiled-coil domain mediating oligomerization to the majority of TRIM family proteins. The C-terminus shares a PRY-SPRY structural domain with some other TRIM family proteins, such as TRIM10, TRIM38, and RNF39, which has been associated with the specific binding of different substrates [25, 26] (Figure **1**). The TRIM26 protein exhibits robust expression in various organs, such as the brain, thymus, lung, liver, spleen, and small intestine, excluding the kidney and heart [27]. It has been demonstrated that the subcellular localization of TRIM26 correlates with the state of viral infection. TRIM26 is localized throughout the cytoplasm when there is no stimulation; however, upon viral infection, a considerable amount of TRIM26 enters the nucleus [27, 28]. This may be mediated by IFN-β, which has experimentally been shown to independently induce nuclear translocation of TRIM26 in HEK293 cells, and Lipopolysaccharide (LPS) was found to have the same effect on HEK293 cells, stably expressing TLR4. In addition, aggregation of TRIM26 in the nucleus was also observed 6-24 hours after HSV-2 infection of VK2 cells [27, 29]. Similar to other proteins of the TRIM family, TRIM26 relies on its RING structural domain to have E3 catalytic enzyme activity and functions to induce specific protein ubiquitination. It is involved in the regulation of various physiological and pathological processes in organisms (Table **1**).

## 3 TRIM26 IN IMMUNITY

Proteins from the TRIM family have demonstrated their crucial involvement in the immune system by participating in various signaling pathways, including the JAK-STAT pathway, TLR-mediated signaling, and dsDNA receptor-mediated signaling [12]. Through decreasing TRAF6 self-ubiquitination and promoting TAB2 degradation, TRIM22 effectively hinders the NF-κB pathway activated by TRAF6 [30]. TRIM11 exerts negative regulation of IFN-β production by targeting TBK1, a key kinase in the RIG-I pathway for phosphorylating IRF3 [31]. Like other TRIM family proteins, TRIM26 also exerts immunomodulatory effects through various mechanisms. Over the past few years, numerous research studies have shown that TRIM26 has the ability to impact viral replication and contribute to inflammatory reactions.

### 3.1 TRIM26 in Viral Infections

The involvement of TRIM26 in viral infections is a subject of debate, and its impact is inconsistently demonstrated in various viral infections or cell cultures. The innate immune response plays a crucial part in the initial defense of the host against viral infection [32]. The body's ability to detect invading viruses is primarily dependent on germ line-encoded Pattern Recognition Receptors (PRRs), which recognize pathogen-associated molecular patterns. This recognition leads to the production of IFNs, proinflammatory cytokines, and downstream interferon-stimulated genes, ultimately establishing an antiviral state [33]. PRRs primarily consist of two categories: Toll-like Receptors (TLRs)-associated receptors that primarily operate in immune cells, such as dendritic cells, macrophages, and B cells, and cytoplasmic RIG-I-like receptors that are widely present in a variety of cells, including LGP2, MDA5, and RIG-I [34]. When the host is infected with an RNA virus, the RIG-I-like receptor recognizes and binds to viral RNA in the cytoplasm, and subsequently, RIG-I and MDA5 are recruited to the mitochondrial junction protein VISA, which serves as a platform for the assembly of a complex that contains multiple signaling proteins, including E3 ligases, such as TRAF2/3/5/6 and TRIM14 [35]. These E3 ligases recruit the NF-κB essential modulator NEMO (also known as IKKg) through self-ubiquitination or mediated ubiquitination of other signaling factors, further recruiting the IKK complex and TBK1/IKKε, which activates Interferon-regulatory Factor 3 (IRF3) and induces the production of IFNs and pro-inflammatory cells [36]. Overexpression and endogenous immunoprecipitation experiments demonstrate that TRIM26 interacts with NEMO at the level of TBK1, and overexpression of TRIM26 enhances this effect in 293T and THP-1 cell lines. TRIM26 may bind to NEMO through self-ubiquitination and polyubiquitination chains, mediate TBK1-NEMO linkage, and dissociate from NEMO after TBK1 is recruited to VISA. Therefore, TRIM26 is involved in the innate immune response against viral infection [32].

**Figure 2:**
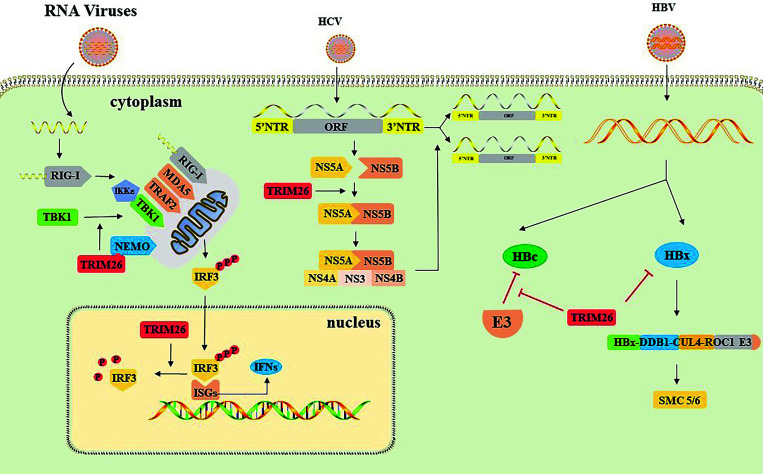
Primary mechanism of TRIM26 in antiviral immunity. TRIM26 mediates TBK1-NEMO linkage by binding to NEMO and dissociates from NEMO after TBK1 is recruited to VISA. TBK1/IKKε activates downstream IRF3 and induces type-I interferon production. TRIM26 binds to part of IRF3 in the nucleus and promotes its polyubiquitination degradation, negatively regulates Interferon-stimulated Genes (ISGs), and reduces IFN-β production. TRIM26 promotes the ubiquitination of NS5B, enhances the binding of NS5B to NS5A, promotes the formation of the HCV gene replication complex consisting of NS5B, NS3, NS4A, NS4B, and NS5A together, and supports viral genome replication. TRIM26 avoids HBc degradation by other E3 ubiquitin ligases, maintains substrate ubiquitination levels, and promotes viral replication. TRIM26 ubiquitinates HBx, inhibits the formation of the HBx-DDB1-CUL4-ROC1 E3 ligase complex, reduces chromosome complex (SMC) 5/6 degradation, and inhibits HBV gene expression.

In contrast, there is evidence suggesting that TRIM26 constitutes one of the termination mechanisms for IRF3 activation [27]. Phosphorylation at multiple phosphorylation sites at the C-terminal of IRF3 mediates the activation of IRF3 into a homodimer that enters the nucleus and binds target genes containing Interferon-stimulated Response Elements (ISREs) to function [37]. Termination of IRF3 activation has been shown to occur by a variety of mechanisms, including dephosphorylation and polyubiquitination [37, 38]. TRIM26 could bind to IRF3 and promote its polyubiquitination and degradation, resulting in a significant reduction in IFN-β production. This ubiquitination occurred in the nucleus, and siRNA knockdown of TRIM26 did not reduce IRF3 expression levels in the cytoplasm. Moreover, this process of ubiquitination exclusively took place between TRIM26 and WT IRF3 as well as the constitutive phosphorylation active mutant 5D. However, the phosphorylation deficient mutant 5A, KR77/78NG, and 5D - KR77/78NG were unable to interact with TRIM26. Validation experiments were performed on macrophages [27] (Figure **2**).

### 3.2 TRIM26 and RNA Viruses

TRIM26 plays a role in human infection with viruses. Firstly, TRIM26 is a key host factor for Hepatitis C Virus (HCV) replication. HCV, a member of the flavivirus genus, is a virus with a single-stranded RNA and an enveloped structure. Its genome is 9.6kb long and consists of an Open Reading Frame (ORF) flanked by highly conserved 5' and 3' untranslated regions. The non-structural proteins NS5B, NS3, NS4A, NS4B, and NS5A encoded by ORF together form an intracellular membrane-associated replication complex responsible for catalyzing viral genome RNA replication. TRIM26 promotes viral gene replication by promoting K27 ubiquitination of NS5B at the K51 site, thereby enhancing its binding to NS5A, and the whole process is not dependent on IFN. This combination is virus-specific and has not been found in other flaviviruses, such as DENV and ZIKV [39].

Selected hosts infected with HIV-1 display encoded antiviral factors that inhibit HIV-1 replication and maintain levels of undetectable viraemia in the absence of antiretroviral therapy; TRIM26 is one of the antiviral factors [40, 41].

In addition to human tissues, the mechanism of antiviral action of TRIM26 has been explored on two porcine cells, PIEC and PAM. On the one hand, polyinosinic:polycytidylic acid [poly (I: C)] stimulation and viral infection (vesicular stomatitis or porcine reproductive and respiratory syndrome virus) can induce porcine TRIM26 expression, thereby evading the host innate antiviral response by inhibiting IFN-β production [42]. On the other hand, ectopic expression of TRIM26 was able to induce degradation of N-protein through binding of the C-terminal PRY/SPRY domain to the N-protein, thus preventing Porcine Reproductive and Respiratory Syndrome Virus (PRRSV) replication [43]. Similar to human infectious viruses, TRIM26 has a dual role in porcine infectious viruses, and more experiments should be conducted on different species and viruses to further clarify the function of TRIM26.

### 3.3 TRIM26 and DNA Viruses

In Hepatitis B Virus (HBV), TRIM26 is able to interact with the HBV core protein (HBc) K7 Lys *via* the SPRY structural domain to avoid HBc degradation and thus promote HBV replication, in contrast to the usual role of E3 ubiquitin ligase performed by TRIM26 [44]. It has been suggested that this may be related to TRIM protein-mediated inactivation of other E3 ligases that maintain substrate ubiquitination levels [45]. Additionally, TRIM26 mutants with a deficiency in the RING structural domain were able to segregate TRIM26-HBc, reversing the process [44]. However, it has also been shown that HBx, a protein encoded by cccDNA (covalently closed circular DNA), is able to bind Damage-specific DNA-binding protein 1 (DDB1) and further form the HBx-DDB1-CUL4-ROC1 E3 ligase complex, which targets chromosomal complex (SMC)5/6 for degradation in the proteasome, thereby enhancing HBV gene expression from cccDNA [46]. The SPRY structural domain of TRIM26 hinders the replication of HBV by ubiquitinating HBx, whereas IFN boosts its anti-HBV impact by increasing the expression of TRIM26. Experiments have been performed on four cell lines: Huh7, HepG2, HepAD38, and HepG2.2.15 [47].

Furthermore, TRIM26 has been demonstrated to inhibit the production of IFN-β and Interferon-stimulated Genes (ISGs) in an immortalized vaginal epithelial cell line (VK2) *via* the IRF3 pathway, thereby facilitating the infection of Herpes Simplex Virus type 2 (HSV-2) and enhancing its replication within the host [29].

### 3.4 TRIM26 in Inflammation

TAK1 plays a crucial role in the activation of NF-κB and the production of pro-inflammatory cytokines during the inflammatory response, triggered by TLRs and cytokines [48]. The activation of TAK1 relies on the clustering of the TAK1-TABs complex, which triggers self or nearby phosphorylation of TAK1. This process is typically believed to be controlled by the conventional protein ubiquitinating enzymes MEKK1 and ITCH [49, 50]. One research work indicated TRIM26 to mediate TAK1 phosphorylation and activation through a non-classical pathway by catalyzing polyubiquitination modification of TAB1 at the K11 junction at Lys294, Lys319, and Lys335, positively regulating the inflammatory immune response. This mechanism has been found to act independently of RNF114 polyubiquitination at the K11 junction [28]. Furthermore, it has been noted that TRIM26 hinders the advancement of nonalcoholic fatty liver disease by suppressing the activation of the C/EBPδ signaling pathway. Specifically, CCAAT/Enhancer Binding Protein Delta CEBPD, which belongs to the C/EBP family of transcription factors, functions as an inflammatory factor that facilitates the release of IL-1β-PTGS2 and activates TLR4 signaling. This ultimately results in the production of TNF-α, MIP-1, and CCL2, as well as the infiltration of inflammatory macrophages. These processes contribute to the development of hepatic steatosis, metabolic changes, insulin resistance, and associated inflammatory reactions [51–54]. TRIM26 works by promoting polyubiquitination degradation of CEBPD in hepatocytes and inhibiting downstream activation of CEBPD-HIF1A signaling [55].

## 4 TRIM26 IN DNA DAMAGE REPAIR

TRIM26 is associated with many aspects of cytogenetic information damage and repair. The endonuclease VIII-like protein 1 (NEIL1), a bifunctional DNA glycosylase, is believed to have the capability of eliminating base damage from single-stranded DNA and bubble structures. It is also involved in repairing particular DNA-damaged substrates, such as telomeric DNA, thereby playing a crucial role in preserving genomic stability [56–59]. It has been demonstrated that the expression level of NEIL1 is regulated by TRIM26 and Mule-mediated ubiquitin-proteasome pathway. *In vitro* experiments showed that TRIM26 and Mule together ubiquitinated the proximal C-terminal Lys of NEIL1 to modulate the cellular DNA damage response [60]. In addition, reactive oxygen species from cellular oxidative metabolism lead to genomic instability by attacking DNA. DNA bases are excised and replaced with the correct undamaged nucleotides in the Base Excision Repair pathway (BER) [61]. Nucleic acid endonuclease III-like protein 1 (NTH1), a protein similar to nucleic acid endonuclease III, is responsible for identifying and eliminating impaired bases, thus initiating the initial phase of BER [62]. TRIM26 was identified as the primary E3 catalase responsible for polyubiquitination of NTH1 in human cell extracts through *in vitro* experiments. This was determined by conducting a ubiquitination activity assay on HeLa whole cell extracts, revealing that TRIM26 acts on lysine 67 of NTH1. In cells, TRIM26 directly polyubiquitinates NTH1 and targets newly synthesized NTH1 proteins for ubiquitination-dependent degradation [63].

## 5 TRIM26 AND SOMATIC CELL REPROGRAMMING

Somatic cell reprogramming is the process by which already differentiated somatic cells are transformed into a pluripotent Embryonic Stem Cell (ESC)-like state under specific conditions using four transcription factors to restore totipotency and generate induced Pluripotent Stem Cells (iPSCs) [64]. iPSCs have the ability to self-renew and differentiate into many different cells, and thus have important roles in disease modelling, drug screening, therapeutic applications, and other fields [65]. A recent study has shown that JMJD3 hinders the process of somatic cell reprogramming through a combination of two mechanisms. On the one hand, JMJD3 promotes cellular senescence and inhibits iPSC reprogramming by regulating promoter H3K27 trimethylation and upregulating p21 and Ink4a; on the other hand, JMJD3 targets Plant Homeodomain Finger protein 20 (PHF20) for K48-linked polyubiquitination and proteasomal degradation in an activity-dependent manner. The identification of PHF20 as a regulator is significant for maintaining the pluripotent state. It not only binds to the Oct4 promoter region, but also interacts with Wdr5 and MOF specifically. Knocking out PHF20 significantly diminished the capacity of Wdr5 and MOF to attach to the promoter of Oct4, suggesting that this protein is critical in reactivating endogenous Oct4 expression and thereby generating fully reprogrammed iPSCs [66].

## 6 TRIM26 IN CANCER

### 6.1 Hepatocellular Carcinoma

It has been shown that TRIM26 is strongly associated with the onset of hepatocellular carcinoma. Initial studies found TRIM26 to be associated with poor outcomes of hepatocellular carcinoma. In comparison to the immortalized normal liver cell line L02, the expression of TRIM26 was reduced in hepatocellular carcinoma samples and several cell lines, such as HepG2, which enhanced hepatocellular carcinoma cell proliferation and clone formation, promoted hepatocellular carcinoma cell proliferation and invasion, as well as affected metabolic reprogramming of tumor cells [67]. Additional research has revealed TRIM26 to maintain the equilibrium of Zinc-finger E-box-binding homeobox 1 (ZEB1) ubiquitination, thereby regulating the advancement of hepatocellular carcinoma through its interaction with the deubiquitinating enzyme USP39. Anomalous Epithelial-mesenchymal Transition (EMT) is regarded as a significant catalyst for tumor infiltration, remote spread, and resistance to medication. ZEB1 is able to induce EMT in tumor epithelial cells. Unlike USP39, which hinders the degradation of ZEB1 by deubiquitination, TRIM26 carries out its cancer-causing role by promoting the degradation of ZEB1 through ubiquitination. USP39 suppressed the shearing and maturation of TRIM26 precursor mRNA, as demonstrated by immunoprecipitation experiments indicating a direct interaction between the two. Simultaneously, they also impacted the EMT procedure through the regulation of β-catenin, a crucial protein in the Wnt/β-catenin signaling pathway [68, 69]. In addition, liver fibrosis is one of the precancerous processes of hepatocellular carcinoma. It has been indicated that mRNA levels of TRIM26 are significantly downregulated in liver fibrosis tissues. Through enhancing the ubiquitination of the Solute Carrier family-7 member-11 (SLC7A11), TRIM26 triggered ferroptosis in Hepatic Stellate Cells (HSC), ultimately reducing liver fibrosis [70].

### 6.2 Lung Cancer

TRIM26 is utilized to a certain degree in the study of Non-small Cell Lung Cancer (NSCLC). The transcription factor PBX1 acts as an inhibitor of NSCLC cell proliferation, clone formation, and survival, and is down-regulated in cancer tissues. However, PBX1 has been reported to have a positive correlation with overall survival in NSCLC patients. The up-regulation of TRIM26 in NSCLC tissues was confirmed by Immunoblotting (IB). TRIM26 functions as an E3 ligase to facilitate the ubiquitination and degradation of PBX1 in the proteasome, thereby promoting tumor growth. This implies that targeting TRIM/PBX1 could potentially be beneficial for NSCLC treatment, although the role of PBX1 in NSCLC requires additional investigation [71]. Interestingly, another study indicated TRIM26 to be lowly expressed in four NSCLC cell lines, including A549, and overexpressed TRIM26 significantly reduced the phosphorylation level of AKT and inhibited the expression level of the cell cycle-related regulators Cyclin D1 and Cyclin D3, suggesting that in NSCLC cells, TRIM26 plays an important role by inhibiting the PI3K/AKT signaling pathway to exert its anti-tumor effect on NSCLC cells [72]. The conclusions are contrary and require further study.

### 6.3 Glioma

In glioblastoma, Glioblastoma Stem-like Cells (GSCs), as a specific subpopulation of tumor cells, show a higher invasive potential, which correlates with overall tumor growth and tumor recurrence after radiotherapy. CDC20-Anaphase-promoting Complex (CDC20-APC) regulates the invasion and self-renewal of human GSCs *via* SOX2 [73]. TRIM26 inhibits the polyubiquitination of SOX2 by WWP2 and prevents its degradation through the PRYSPRY structural domain located at its C-terminal. This stabilization of SOX2 protein helps to maintain the tumorigenic capacity of GSCs, regardless of its RING structural domain [45]. Studies have demonstrated that TRIM26 is involved in gliomas by facilitating the K63-linked polyubiquitination of GPX4, a cellular agent that safeguards against ferroptosis. This process takes place at the K107 and K117 sites, counteracting its K48-linked polyubiquitination and ensuring that the GPX4 protein remains stable. Also, in this study, PLK1-mediated S127 phosphorylation of TRIM26 was required during the interaction of TRIM26 with GPX4. Thus, the knockdown of TRIM26 has been found to induce ferroptosis in glioma cells and inhibit their tumorigenic capacity [74].

### 6.4 Other Cancers

TRIM26 has also been extensively documented in various other types of cancers. The levels of p-Akt, p-GSK3β, β-catenin, and c-Myc were notably decreased in T24 and J82 cells with TRIM26 knockdown in bladder cancer, resulting in the suppression of tumor cell proliferation, migration, and invasion. In T24 and J82 cells transfected with sh2-TRIM26, the Akt activator SC79 effectively counteracted the reduction in cell proliferation caused by TRIM26 knockdown. Moreover, it notably enhanced the levels of p-Akt, pGSK3β, β-catenin, and c-Myc expression. Moreover, knockdown of β-catenin eliminated the cell migration and invasion induced by TRIM26 [75]. Research has demonstrated that when AKT is activated, it phosphorylates GSK-3β, leading to its inactivation. Activation of downstream glycogen synthase and accumulation of β-catenin in the cytoplasm establish a significant connection between the PI3K/Akt pathway and the Wnt/β-catenin pathway [76]. Therefore, TRIM26 regulates cell proliferation, migration, and invasion through the AKT/GSK3β/β-catenin pathway [75].

In addition, DNA sequence polymorphisms known as Single Nucleotide Polymorphisms (SNP) arise from variation in a single nucleotide at the genomic level. Research has revealed that the variant of SNP, rs117565607, plays a role in controlling the expression of TRIM26 in nasopharyngeal carcinoma. Furthermore, NPC tissue samples with a chain genotype of AA/AT exhibit a notable decrease in TRIM26 expression. Further investigation revealed decreased expression of immune-related genes, such as NFKB2, IL-32, IRF7, CD38, and STAT1 upon silencing of TRIM26, indicating that the suppression of TRIM26 is linked to a diminished immune reaction in NPC. Meanwhile, down-regulated TRIM26 directly attenuated the cytotoxicity and tumor-killing susceptibility of NK cells, thus supporting a mechanism related to immune escape in nasopharyngeal carcinoma with low TRIM26 expression [77].

In Papillary Thyroid Carcinoma (PTC), TRIM26 has been reported to be significantly down-regulated in tissues and cell lines, and overexpression of TRIM26 has been reported to significantly inhibit the phenotypes of PTC cells, such as proliferation, migration, and EMT, as well as the activation of glycolysis and PI3K/Akt signaling pathways [78]. In osteosarcoma, a comparable phenotypic occurrence takes place, although it varies from PTC in the sense that overexpressed TRIM26 obstructs the growth and invasion of cells by attaching to and breaking down RACK1, consequently deactivating the MEK/ERK pathway. In addition, whether RACK1 mediates the effects on the MEK/ERK pathway through the Protein Kinase C (PKC) signaling pathway needs to be further explored [79]. In endometrial cancer, TRIM26 inhibits cancer growth by regulating the AKT pathway and apoptotic processes and correlates with survival indicators [80]. Based on the analysis of online databases, it has been observed that TRIM26 exhibits low expression levels in renal clear cell carcinoma, which is linked to reduced survival rates. However, additional experimental and clinical data are required to confirm these findings [81].

## 7 TRIM26 IN NEUROLOGICAL DISORDERS

Spina bifida (Sb) in the fetus is a prevalent congenital anomaly in humans, typically resulting from the incomplete merging of the neural tube at the lower end. Maternal periconceptional folic acid supplementation can prevent 70% of Sb cases [82]. The placenta acts as an intermediary, capable of translating information about its genetic changes directly to the fetus. According to research, there is a significant correlation between abnormal DNA methylation in the placenta and the pathological characteristics of fetal Sb. Initially, the DNA methylation microarray analysis indicated that the quantity of hypomethylated regions in the Sb placenta exceeded the quantity of hypermethylated regions. Western blotting demonstrated that TRIM26 protein levels were significantly higher in Sb placentas than in normal controls, while protein expression of TRIM26 was significantly higher after treatment of cell lines with the DNA methyltransferase inhibitor 5-aza-2′-deoxycytidine. In addition, the protein level of TRIM26 was higher in cells deficient in dihydrofolate reductase than in normal cells. These studies suggest that TRIM26 may be associated with folate deficiency and placental DNA hypomethylation levels in fetal spina bifida [83]. In another study, TRIM26 was suggested to be associated with a potential relationship with AS3MT and thus induce epilepsy [84]. In a separate group of individuals with schizophrenia (n = 202), TRIM26 exhibited differential expression and is regarded as a potential gene associated with schizophrenia [85].

## 8 DISCUSSION

In the past decades, ubiquitination as one of the ways of post-translational modification of proteins has been widely noticed and studied. Researchers have also observed TRIM26, which is a part of the E3 ubiquitin ligase group.

On the one hand, the study of TRIM26 can further unravel the mechanism of development of various diseases and provide new targets for precision therapy; on the other hand, implanting human TRIM26 into animal models may improve their tolerance to viral infection and provide new methods for disease modeling.

However, there is still a great deal of space for exploration in understanding the structure and function of TRIM26. TRIM26 shows potential value in neurological diseases. A large number of neurodegenerative diseases are triggered by normal protein aggregation produced at the physiological level. It has been shown that TRIM family proteins usually function as a disaggregase, mediating the disruption of misfolded aggregated proteins, and the potential of TRIM26 in this regard is not yet known [20]. Coincidentally, in T2DM, glycated proteins also form amyloid entities, which may trigger cognitive deficits, yet this glycation happens to occur on Lys, consistent with a TRIM-mediated ubiquitin molecule-substrate linkage site of action [86]. Whether this substrate-level protein glycation attenuates this disaggregase action deserves further exploration.

In the available studies on inflammation, we have noticed that TRIM26 achieves its regulation of the inflammatory immune response through the downstream NF-κB pathway and C/EBP [28, 55]. Regulatory T cells (Tregs) are a subpopulation of T cells with negative immunomodulatory functions that suppress the immune response of other immune cells. Interestingly, it has been reported in the literature that both the NF-κB pathway and C/EBP are able to drive Treg cell development by promoting FOXP3 (Forkhead box P3) expression for inflammatory responses [87, 88]. It is worth pondering whether the NF-κB pathway and C/EBP activated by TRIM26 are related to the development of Treg and whether the anti-inflammatory effects of Treg synergise with TRIM26.

In addition, Intrinsically Disordered Regions (IDRs) are prevalent in proteins, and these protein fragments do not have a fixed three-dimensional structure. Recent studies have suggested that mutations occurring on IDRs lead to chromatin remodeling and dysregulated gene expression [89]. Prediction of the TRIM26 structure *via* the online site PSIPRED revealed that the IDRs of TRIM26 may occur at amino acids 126-131, and that these IDRs may be a future research direction (Fig. **S1**). The expression of the TRIM26 protein and the downstream signaling pathways activated by TRIM26 vary in different tumors, and the types of tumors involved are not sufficiently wide-ranging. So far, there are also no drugs developed against TRIM26 targets.

## CONCLUSION

This article has provided a comprehensive review on the composition and operation of TRIM26 and its participation in numerous biological and pathological procedures. TRIM26 is implicated in aspects of immunity, inflammation, and tumor development. In terms of viral infection, TRIM26 has a dual role, showing promotion or inhibition of viral replication in different situations, respectively, and the opposite conclusion may be attributed to different viral species and hosts, or the heterogeneity of the target cells, which deserves further exploration. Additionally, TRIM26 has the ability to penetrate the nucleus and engage in activities, like repairing DNA damage and reprogramming somatic cells. Overall, TRIM26 is considered to be one of the essential regulators in various biological activities, and its potential role and mechanism deserve further investigation.

## Figures and Tables

**Table 1:** Mechanisms of TRIM26 substrate interactions and pathophysiological processes involved.

**Substrate**	**Pathological or Physiological Processes**	**Mechanism**	**Impact**	**References**
NEMO	RNA virus infections	TRIM26 binds to the NF-kB key factor NEMO *via* self-ubiquitination and polyubiquitination chains, mediates TBK1-NEMO linkage, and dissociates from NEMO after TBK1 is recruited to VISA	The VISA complex recruits the IKK complex and TBK1/IKKε, which activates IRF3 and induces the production of type I IFNs and pro-inflammatory cells	[32]
IRF3	RNA virus infections	TRIM26 binds to wild-type IRF3 and mutant 5D, promoting their polyubiquitination and degradation	IFN-β production is reduced, which negatively regulates Interferon-stimulated Genes (ISGs) and increases Herpes Simplex Virus type-2 (HSV-2) replication in the host	[27, 29]
NS5B	Hepatitis C virus infection	TRIM26 promotes K27 ubiquitination of NS5B at the K51 site, enhances the binding of NS5B to NS5A, and facilitates the formation of the HCV gene replication complex consisting of NS5B, NS3, NS4A, NS4B, and NS5A, which is IFN-independent	It acts as a viral replication host factor to support viral genome replication	[39]
HBc	Hepatitis B virus infection	TRIM26 interacts with the K7 Lys of the HBV core protein (HBc) through the SPRY domain, preventing HBc from being degraded by other E3 ubiquitin ligases	Substrate ubiquitination levels are maintained and viral replication is promoted	[44]
HBx	Hepatitis B virus infection	TRIM26 ubiquitinates HBx through the SPRY domain, inhibiting the formation of the HBx-DDB1-CUL4-ROC1 E3 ligase complex and reducing the degradation of chromosomal complex (SMC) 5/6 in the proteasome	Inhibits HBV gene expression from cccDNA	[47]
Unknown	HIV-1 infection	Unknown	It acts as an antiviral factor to inhibit HIV-1 replication and maintain undetectable levels of host viraemia	[40, 41]
IFN-β	VSV, PRRSV infection	TRIM26 inhibits IFN-β production	Evades innate host antiviral response	[42, 43]
N protein	PRRSV infection	TRIM26 binds to and promotes degradation of N proteins *via* the C-terminal PRY/SPRY structural domains	PRRSV replication is blocked	[43]
TAK1	Inflammatory response	TRIM26 mediates TAK1 phosphorylation and activation through a non-classical pathway catalyzing polyubiquitination modification of the K11 linkage of TAB1 at Lys294, Lys319, and Lys335	It activates NF-κB and produces pro-inflammatory cytokines	[28, 48]
CEBPD	Steatohepatitis	TRIM26 promotes polyubiquitination and degradation of CEBPD in hepatocytes, reduces IL-1β-PTGS2 release, and inhibits TLR4 signaling activation, suppressing TNF-α, MIP- 1, and CCL2 production, and macrophage inflammatory infiltration	It promotes hepatic steatosis, metabolic changes, insulin resistance, and related inflammatory responses	[55]
NEIL1	Reparation of DNA damage	TRIM26 and Mule ubiquitinate the near C-terminal Lys of NEIL1 and reduce intracellular NEIL1 levels	Increases cellular sensitivity to ionising radiation and hydrogen peroxide-induced cell death	[60]
NTH1	Reparation of DNA damage	TRIM26 catalyzes polyubiquitination degradation of NTH1 at lysine 67	Reduces oxidative DNA damage repair capacity and resistance to hydrogen peroxide-induced cell killing	[63]
PHF20	Somatic cell reprogramming	JMJD3 targets PHF20 for K48-linked polyubiquitination and proteasomal degradation by recruiting TRIM26 in a non-demethylase activity-dependent manner	It induces a pluripotent embryonic stem cell-like state in somatic cells, transforming them into pluripotent stem cells	[66]
ZEB1	Hepatocellular carcinoma	TRIM26 ubiquitinates the degradation of ZEB1 and regulates β-catenin, a key protein in the Wnt/β-catenin signaling pathway	It inhibits aberrant Epithelial-mesenchymal Transition (EMT) and tumor progression	[68]
SLC7A11	Liver fibrosis	TRIM26 promotes ubiquitination of the Solute Carrier family-7 member-11 (SLC7A11), a key protein in ferroptosis	It induces iron ferroptosis in Hepatic Stellate Cells (HSC) and attenuates hepatic fibrosis	[70]
PBX1	Non-small cell lung cancer	TRIM26 mediates ubiquitination and degradation of PBX1 in the proteasome	It promotes tumor proliferation	[71]
AKT	Non-small cell lung cancer	TRIM26 reduces the expression level of cell cycle-related regulators cyclin D1 and cyclin D3 by inhibiting the PI3K/AKT signaling pathway	It inhibits tumor proliferation	[72]
Unknown	Bladder cancer	TRIM26 activates AKT/GSK3β/β-catenin pathway	It promotes cell proliferation, migration, and invasion	[75]
SOX2	Glioma	TRIM26 competes with WWP2 for binding to the N-terminus of SOX2, thereby inhibiting WWP2-mediated SOX2 polyubiquitination and subsequent proteasomal degradation, and stabilizing SOX2 protein	It maintains the tumorigenic capacity of glioblastoma stem-like cells	[45, 73]
GPX4	Glioma	TRIM26 catalyzes the K63-linked polyubiquitination of the cellular ferroptosis protector GPX4 at the K107 and K117 loci and reverses its K48-linked polyubiquitination, thereby maintaining GPX4 protein stability	It induces ferroptosis and inhibits tumorigenicity of glioma cells	[74]
IRF7, NFKB2, NK cell	Nasopharyngeal cancer	The A allele of SNP rs117565607 mediates the down-regulation of TRIM26, which decreases the expression of IFN-mediated immunity genes, including IRF7 and NFKB2, and directly attenuates the cytotoxicity and tumor-killing susceptibility of NK cells	Low expression of TRIM26 enhances immune escape of nasopharyngeal carcinoma cells	[77]
Unknown	Thyroid cancer	TRIM26 activates glycolysis and the PI3K/Akt signaling pathway	It inhibits PTC cell proliferation, migration, and EMT phenotypes	[78]
RACK1	Osteosarcoma	TRIM26 binds and degrades RACK1 and inactivates the MEK/ERK pathway	It inhibits cell proliferation and invasion	[79]

## LIST OF ABBREVIATIONS

BER: Base Excision Repair
CC: Coiled Coil
cccDNA: Covalently Closed Circular DNA
CDC20-APC: CDC20-anaphase-promoting Complex
DDB1: Damage-specific DNA-binding Protein 1
EMT: Epithelial-mesenchymal Transition
ESC: Embryonic Stem Cell
FOXP3: Forkhead Box P3
GSCs: Glioblastoma Stem Cell-like Cells
HBc: HBV Core Protein
HBV: Hepatitis B Virus
HCV: Hepatitis C Virus
HSC: Hepatic Stellate Cell
HSV-2: Herpes Simplex Virus Type 2
IB: Immunoblotting
IDRs: Intrinsically Disordered Regions
IFN: Interferon
iPSCs: Induced Pluripotent Stem Cells
IRF3: Interferon-regulatory Factor 3
ISGs: Interferon-stimulated Genes
ISREs: Interferon-stimulated Response Elements
LPS: Lipopolysaccharide
MHC: Major Histocompatibility Complex
NEIL1: Endonuclease VIII-like Protein 1
NS1: Nonstructural Protein 1
NSCLC: Non-small Cell Lung Cancer
NTH1: Nucleic Acid Endonuclease III-like Protein 1
ORF: Open Reading Frame
PAMPs: Pathogen-associated Molecular Patterns
PBX1: Pre-B Cell Leukemia Transcription Factor 1
PHF20: Plant Homeodomain Finger Protein 20
PINK1: PTEN-induced Putative Kinase 1
PKC: Protein Kinase C
Poly (I:C): Polyinosinic:polycytidylic Acid
PRRs: Pattern Recognition Receptors
PRRSV: Porcine Reproductive and Respiratory Syndrome Virus
PTC: Papillary Thyroid Carcinoma
Sb: Spina Bifida
SLC7A11: Solute Carrier Family-7 member-11
SNP: Single Nucleotide Polymorphisms
TAK1: Transforming Growth Factor-β Activated Kinase-1
TLRs: Toll-like Receptors
Treg: Regulatory T Cells
TRIM: Tripartite Motif-containing
VSV: Vesicular Stomatitis Virus
ZEB1: Zinc-finger E-box-Binding Homeobox 1
