# Supplementary material for: The Multifunction of TRIM26: From Immune Regulation to Oncology
Source: Protein Pept Lett. 2024 Jul 2;31(6):424–36. doi: 10.2174/0109298665311516240621114519 (PMC11475100; doi:10.2174/0109298665311516240621114519)
Supplement: Supplementary file 1 [file PPL-31-424_SD1.pdf]

Supplementary Materials

The Multifunction of TRIM26: From Immune Regulation to Oncology

Jialai Zou[1,#], Kaiyi Niu[1,#], Tao Lu[1], Jianxun Kan[1], Hao Cheng[1] and Lijian Xu[1,*]

[1]*Department of General Surgery, The Second Affiliated Hospital of Nanjing Medical University, Nanjing 210011, China*

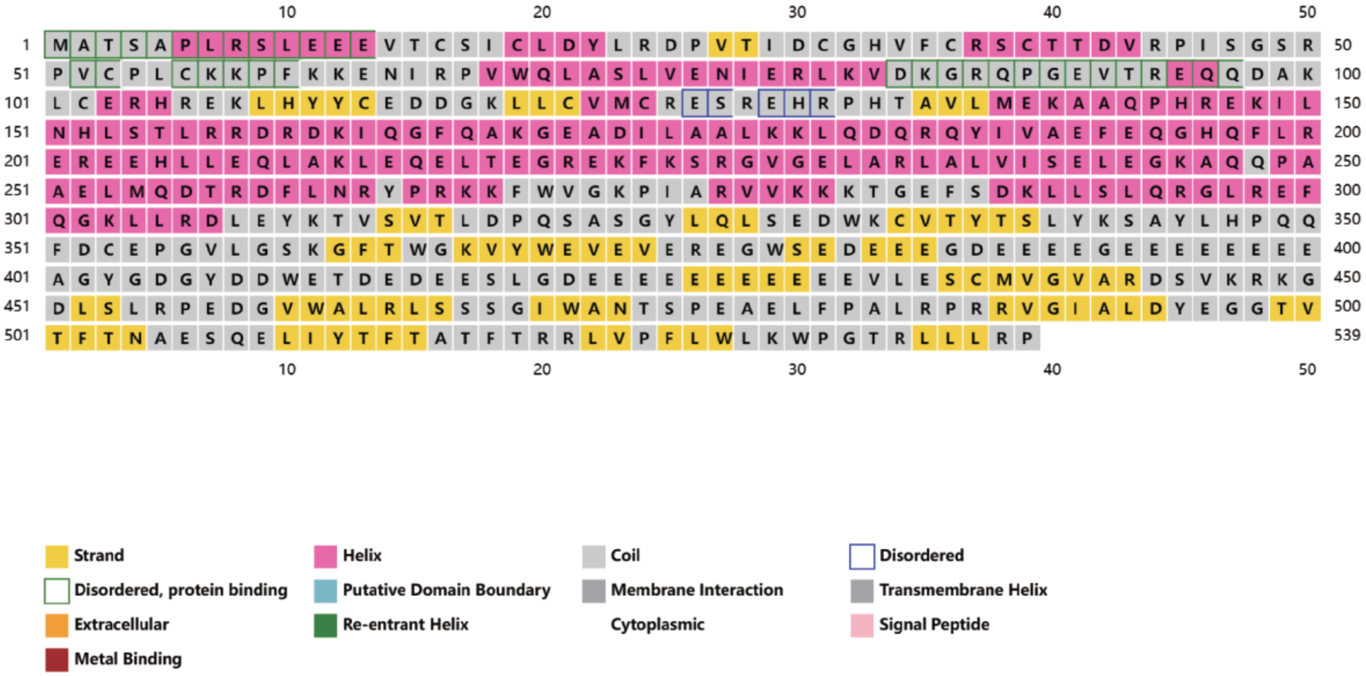

Figure S1.

 © 2024 Bentham Science Publishers
